# Chagas disease diagnosis and cure assessment: Getting formally hierarchical about a naturally hierarchical problem

**DOI:** 10.1371/journal.pntd.0008751

**Published:** 2020-10-29

**Authors:** Fernando Abad-Franch

**Affiliations:** Núcleo de Medicina Tropical, Faculdade de Medicina, Universidade de Brasília, Brasília, Brazil; Tulane University, UNITED STATES

## Detecting pathogens in infected hosts: An informal account

In a recent Viewpoint article, Alonso-Padilla and colleagues [1] discuss the difficulties of confidently classifying a patient with Chagas disease as “cured”—with “cure” defined as the “elimination of *Trypanosoma cruzi* parasites from the patient’s body following treatment” [1]. This is, of course, a particular instance of a much more general problem: doctors need to confidently classify subjects as infected or not with a pathogen to make clinical decisions, to understand infectious disease epidemiology, and to measure the effects of therapeutic or preventive interventions.

Yet detecting pathogens in infected bodies is typically far from easy. One common setback is that the pathogen may be present in some parts of the subject’s body (so that *the subject is infected*), yet absent from the specific *sample* used for testing—say, a drop of blood. In the case of Chagas disease, *T*. *cruzi* is indeed absent from the bloodstream of infected subjects most of the time, particularly in the chronic phase, and bloodstream parasites should become even rarer after patients take parasite-killing drugs. A negative test result when the target pathogen was *not available for detection* in the tested sample can hardly be seen as a “true” false negative—the test gave the *correct* answer at the *sample* level, notwithstanding the fact that the subject *was* infected. (Note that the detection “target” may be the pathogen itself or a surrogate biomarker—whether pathogen-derived, such as proteins or nucleic acids, or host-derived, such as antibodies [2–7].) Unfortunately, a test can also yield a negative result despite the target being present, and hence *available for detection*, in a given sample from an infected subject; this qualifies as a *true* false negative. In practice, *any* test can fail to detect a target pathogen (or biomarker) that is present in a sample because all tests have imperfect (<1.0) sensitivity. Finally, a test may yield a *positive* result out of a sample that did *not* contain the target. This reflects the general problem of imperfect test specificity. In what follows, I will focus on target *availability* and test *sensitivity* by temporarily assuming 100% specificity; although hard to ensure even under best sampling and laboratory practices, specificity may be close to 100% for direct ascertainment of pathogen presence (e.g., through microscopy-, culture-, or xenodiagnosis-based parasitological methods) and for some DNA-based tests (e.g., using PCR or isothermal amplification) (see [2,3,6] and, for an example on *T*. *cruzi* detection in its vectors, [8]). I will revisit imperfect specificity at the end of my argument.

**Fig 1:**
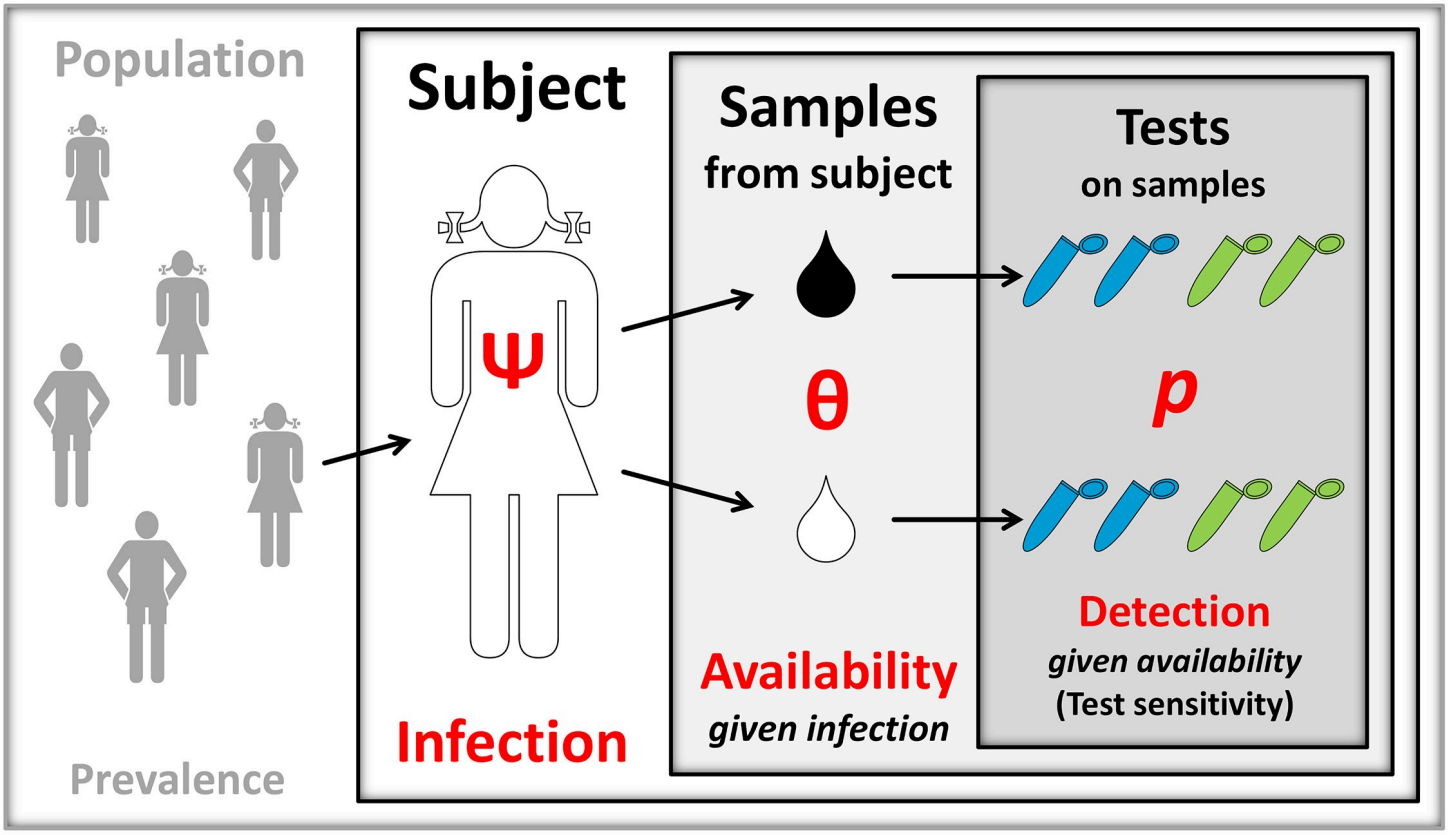
The biologically hierarchical problem of detecting a pathogen in its hosts. A *subject* drawn from a population is infected with the pathogen with probability Ψ (which reflects the prevalence of infection in that population). A *sample* drawn from a subject contains the pathogen with probability θ, which is conditional on Ψ. Therefore, θ represents sample-level pathogen (or, more broadly, target) *availability*, given subject-level infection. Finally, pathogen detection *tests* run on samples drawn from a subject detect the target with a probability *p* that is conditional on θ; therefore, *p* represents test-level probability of *detection*, given sample-level availability (and, hence, subject-level infection). This shows that the biological problem of detecting a pathogen in its hosts involves a hierarchy of nested levels: subjects (within populations), samples within subjects, and tests within samples. To understand how the system works, we therefore need to estimate 3 probabilities: test-level detection (*p*), sample-level availability (θ), and subject-level infection (Ψ). Replicate tests inform on the value of *p* (which is, strictly speaking, the sensitivity of the test) and replicate samples (possibly encompassing different tissues or bodily fluids, as suggested by black/white drops) drawn from the same subject inform on the value of θ; this opens the possibility of estimating Ψ and, therefore, prevalence in the population. Note that all the parameters above (Ψ, θ, and *p*) are probabilities and hence can take on any value between 0 and 1.

The previous paragraph contains an informal account of the problem of *detecting a target pathogen (or surrogate biomarker) in samples drawn from human subjects*. It suggests that there are a few levels at which different things happen with different, albeit related, probabilities. Thus, a *subject* from a population may (or may not) be *infected* with a pathogen; a *sample* from an infected subject may (or may not) *contain* the target pathogen/biomarker; and a *test* may (or may not) *detect* the target in a sample that comes from an infected subject and contains that target (Fig 1). This description of the problem also suggests that the performance of a test can only be properly assessed by specifying what level the “answer” of the test (“positive” or “negative”) refers to—the very same test result may well be “correct” at one level and “incorrect” at another. For example, a negative test result can be correct at the *sample* level if the target was unavailable for detection in that sample but incorrect at the *subject* level if that subject was infected. This informal account is, in my view, fairly instructive, but I think that it can be much more useful if translated into a formal statement of the problem.

## A formal statement of the hierarchical problem

Note, first, that the 3 levels mentioned above are naturally arranged into a nested *hierarchy* (Fig 1). On top of the hierarchy, subjects are infected with a certain probability (denoted Ψ). At the next level, samples drawn from subjects contain the target pathogen (or biomarker) with a certain probability (denoted θ) that is *conditional* on the subject being infected. Finally, a test detects the target pathogen/biomarker in a sample with a certain probability (denoted *p*) that is *conditional* on the target being available for detection in the sample (which must therefore come from an infected subject) (Fig 1). Thus, Ψ measures subject-level *infection* (and, under certain sampling designs, provides an estimate of population-level *prevalence*); θ measures sample-level *availability* of the target, given infection; and *p* measures test-level *sensitivity*, given infection and availability (Fig 1). If doctors read a “negative” result from a test run on a sample drawn from a randomly chosen subject of unknown infection status, they must interpret this observation as arising from one of three possibilities:

1. The subject was *not infected* (with associated probability 1−Ψ); or
2. The subject was infected (with probability Ψ), yet the target pathogen/biomarker was *not available* for detection in the sample (with probability 1−θ); or
3. The subject was infected (with probability Ψ), and the target was available for detection in the sample (with probability θ), yet it was *not detected* by the test (with associated probability 1−*p*).

Formally, then, the probability of observing a negative test result (“0”) is

Pr(0)=(1−Ψ)+Ψ×(1−θ)+Ψ×θ×(1−p),

where each additive term represents one of the three possibilities laid out above. If the doctors read a positive test result, then they know (assuming 100% specificity for the moment) that the subject was *infected*, and the target pathogen/biomarker was *available* for detection in the sample and the test *detected* it. The probability of observing a positive test result (“1”) is Pr(1) = Ψ × θ × *p*. This shows that the observed probability of getting a positive result when testing an infected subject (with Ψ = 1.0) is the product of 2 probabilities (θ × *p*), a mix for which “test sensitivity” is a misnomer. This value will *only* equal the true sensitivity of the test (*p*) if θ = 1.0—that is, if we can assume that the target is *always* available in any sample taken from an infected subject. But θ is seldom equal to 1.0. Average θ is clearly <1.0 for *T*. *cruzi* parasites and their DNA in blood samples; it may approach 1.0 for certain antibody classes such as immunoglobulin G (IgG) in chronically infected subjects (often with the downside of low specificity) and possibly for some pathogen-derived biomarkers [1–6], and can only be 1.0 when the target was seeded in the sample by the investigators—that is, in the artificial context of laboratory studies designed to determine analytical sensitivity [6,7].

It is, I believe, very important to realize that what many, if not most, field studies report as “test sensitivity” is, in reality, θ × *p*, and that this entails a strong, implicit, and often unjustified assumption that sample-level availability is perfect (θ = 1.0). Reporting θ × *p* as a single figure hardly helps us understand how the system actually works—a “0.5” value is as compatible with low sample-level availability and high test-level sensitivity (say, θ = 0.51 and *p* = 0.98) as it is with the opposite (say, θ = 0.93 and *p* = 0.54). How can we tackle these issues in the real-life settings of epidemiological or clinical research practice? I suggest that one potentially fruitful starting point is to borrow insight from wildlife ecology studies that deal with problems involving a hierarchy of related probabilities analogous to that depicted in Fig 1.

### A formally hierarchical approach to a naturally hierarchical problem

Note, first, that the problem of whether a wild animal occupies a given *habitat patch* is precisely analogous to the problem of whether a pathogen infects a given *subject*. Second, the problem of whether an animal that occupies a habitat patch is available for detection at a given *sampling point* within that patch is analogous to the problem of whether a pathogen that infects a subject is available for detection in a given *sample* drawn from that subject. And, third, the problem of whether a given *detection method* detects an animal that occupies a habitat patch and is available for detection at a sampling point within that patch is analogous to the problem of whether a given *test* detects a pathogen that infects a subject and is available for detection in a sample drawn from that subject.

Wildlife ecologists have developed a suite of hierarchical modeling approaches to tackle hierarchical problems of this kind [9,10]. They use repeated sampling (in a version of Pollock’s “robust design” [11]) to gather the information they need to estimate, under a set of assumptions, the parameters of interest (habitat patch-level occupancy, Ψ; sampling site-level availability, θ; and detection method-level sensitivity, *p*) and their variances [10]. Repeated sampling entails making several independent observations at each level of the hierarchy—more than a single habitat patch, more than a single sampling site within each habitat patch, and more than a single attempt at detection within each sampling site. The analogy now goes: take more than a single sample from more than a single subject, and run more than a single test on each sample.

**Fig 2:**
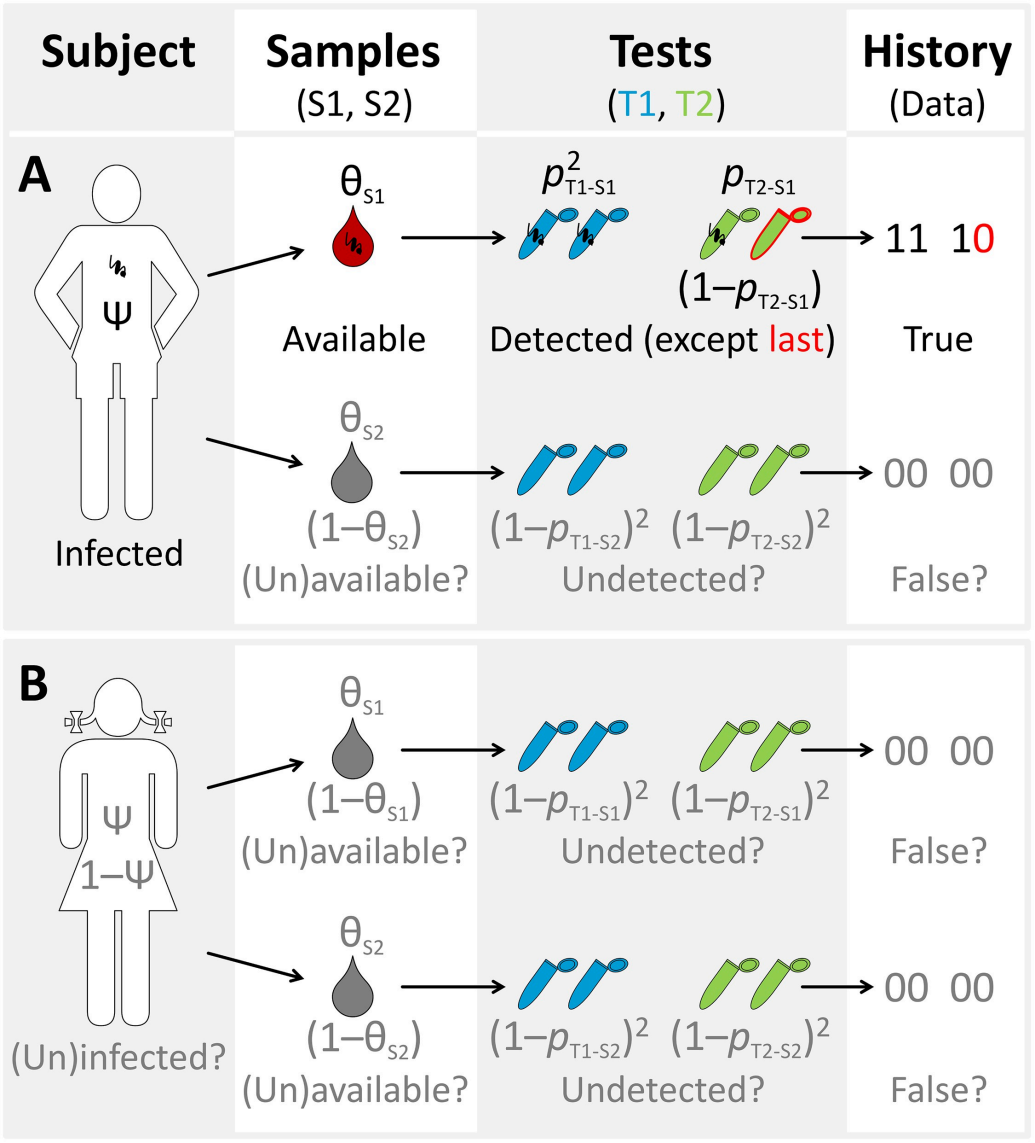
Detecting *Trypanosoma cruzi* in human subjects: A hierarchical approach to a hierarchical problem. Two tests (T1 and T2) are run in duplicate on each of 2 samples (S1 and S2) drawn from subjects A and B; the observed data come in the form of a “history” of detection (coded “1”) or non-detection (“0”) of the parasite (or a surrogate biomarker). In **A**, both T1 and the first T2 (but not the second) replicates run on S1, and none of the 4 run on S2, yielded a “1.” This means (assuming no false positives) that *T*. *cruzi* (represented by a small curly object) (i) was infecting subject A (Ψ); (ii) was available for detection in S1 (red drop; θ_S1_) and indeed detected twice by T1 (*p*_T1-S1_ × *p*_T1-S1_) and once by T2 (*p*_T2-S1_), yet the second T2 failed to detect it (1−*p*_T2-S1_) (this is, hence, a true false negative; highlighted in red); and (iii) was not detected by any test in S2—which may be because *T*. *cruzi* was unavailable for detection in S2 (1−θ_S2_) or because it was available (θ_S2_), yet all test failed to detect it (1−*p*_T1-S2_)^2^ × (1−*p*_T2-S2_)^2^. I use gray font to highlight ambiguities. Was the parasite available in S2, but went undetected? Or was it unavailable, so that the tests gave the correct (sample-level) answer? Are the “0”s from S2 true negatives, or are they false negatives? In **B**, no detections are recorded in the subject’s history, and all observations are therefore ambiguous. Was the subject infected? If she was infected, was the parasite unavailable in both samples? Or was it available, yet the tests failed to detect it? Are the “0”s true or false negatives? Note (i) that replicate samples may be taken from more than a single tissue or bodily fluid, as well as on more than 1 occasion, and (ii) that all the parameters above (Ψ, θ, and *p*) are probabilities and hence can take on any value between 0 and 1.

In the case of *T*. *cruzi*, for example, one might take 2 blood samples (e.g., on 2 consecutive days) from each subject and run 2 duplicate PCRs (4 reactions), perhaps each targeting a different DNA stretch, on each sample. If a pathogen (or biomarker) may occur in several fluids or tissues (saliva, tears, urine, blood…), one might collect samples of those fluids or tissues and run replicate tests on each. To preserve independence, each test must be run and scored without knowing the outcome of other tests or the infection status of the subject—ideally, the person running the test and scoring it as positive or negative should be blinded to subject and sample identity. The outcome of this sampling/testing design is a “history of detection” of the target pathogen (or biomarker) in replicate tests run on replicate samples from each subject. For example, doctors might get, for subject A, the following outcomes (Fig 2A):

1. Subject A/Sample 1/Test 1: both replicates positive (coded “11”)
2. Subject A/Sample 1/Test 2: one replicate positive, the other negative (coded “10”)
3. Subject A/Sample 2/Test 1: both replicates negative (coded “00”)
4. Subject A/Sample 2/Test 2: both replicates negative (coded “00”)

or, in short, “11 10 00 00.” What do the doctors learn from these observations? First, the subject *was infected* (with associated probability Ψ). Second, the target *was available* for detection in sample 1 (with associated probability θ_S1_) and *was detected* twice by test 1 (*p*_T1-S1_ × *p*_T1-S1_) but only once by test 2 (*p*_T2-S1_ × (1−*p*_T2-S1_)). And, third, the target was *not detected* by any of the 4 tests run on sample 2; this may be either because the target was not available in that sample (1−θ_S2_) or because all tests failed to detect it even though it was available (θ_S2_ × (1−*p*_T1-S2_)^2^ × (1−*p*_T2-S2_)^2^). All in all, the probability of observing subject A’s detection history is

$$Pr(11\;10\;00\;00)=\Psi \times [\theta_{S1}\times p_{T1‐S1}\times p_{T1‐S1}\times p_{T2‐S1}\times (1-p_{T2‐S1})]\times [(1-\theta_{S2})+\theta_{S2}\times (1-p_{T1‐S2})\times (1-p_{T1‐S2})\times (1-p_{T2‐S2})\times (1-p_{T2‐S2})].$$

If, for subject B, all test replicates were negative (Fig 2B), we would have

$$\begin{array}{l} Pr(00\;00\;00\;00)=\\ \left(1-\Psi \right)+\\ \Psi \times (1-\theta_{S1})\times (1-\theta_{S2})+\\ \Psi \times \theta_{S1}\times {\left(1-p_{T1‐S1}\right)}^{2}\times {\left(1-p_{T2‐S1}\right)}^{2}\times (1-\theta_{S2})+\\ \Psi \times \theta_{S2}\times {\left(1-p_{T1‐S2}\right)}^{2}\times {\left(1-p_{T2‐S2}\right)}^{2}\times (1-\theta_{S1})+\\ \Psi \times \theta_{S1}\times \theta_{S2}\times {\left(1-p_{T1}\right)}^{4}\times {\left(1-p_{T2}\right)}^{4}. \end{array}$$

That is, either subject B was not infected (first additive term) or she was infected but (i) the target pathogen/biomarker was unavailable in both samples (second term), or (ii) the target was available in sample 1 but went undetected by all 4 PCR replicates and was not available in sample 2 (third term), or (iii) the target was available in sample 2 but went undetected by all 4 PCR replicates and was not available in sample 1 (fourth term), or (iv) the target was available in both samples but went undetected by all 8 PCR replicates (fifth additive term) (Fig 2B).

Assuming subjects are independent, the likelihood of the whole set of observations is the product of probabilities over all subject-specific detection histories—and one can estimate the values of the parameters of interest that maximize the likelihood [9,10]. With enough independent data and a generalized linear modeling framework, it is also possible to investigate the effects of subject, sample, or test covariates on Ψ, θ, and *p* [9,10]. Inference can first be based on comparing the performance of different model specifications, each representing an explicit hypothesis about how the system works. Relative model performance can be measured using, e.g., Akaike’s information criterion corrected for small samples (AICc) and Akaike model weights [12]. For example, one may use this approach to gauge the relative support for the assumption of perfect availability (θ = 1.0) by comparing a model in which θ is estimated from the data with an alternative model in which the value of θ is fixed at 1.0. Or one may compare a model in which θ is constant across subjects with another in which θ is allowed to vary depending on whether each subject was treated or not—in the expectation that treatment will reduce bloodstream parasite availability. Quantitative inference can then proceed by either focusing on the coefficient estimates and predictions of the top-performing (smallest-AICc) model or, if no individual model clearly outperforms the rest (i.e., differences in AICc scores are small—say, <4.0), on model-averaged estimates—all with appropriate measures of uncertainty [9,10,12].

As noted above, an assumption of this approach is that the tests have 100% specificity—there are no false-positive results in the data set. Models that accommodate both the three-level hierarchy discussed here (subject, sample, test) and the possibility of false-positive results are “structurally unidentifiable”—different sets of parameter values fit the data equally well. To solve this issue, one needs both ancillary information on false-positive detection rates (e.g., from calibration experiments with negative and blank controls) and additional results from a method that yields unambiguous detections (e.g., direct visualization of pathogen presence by microscopy) [13,14]. In practice, the wisest tactic is to minimize the possibility of getting false positives by using highly specific targets (which may involve PCR primer design or antigen selection, especially for emerging pathogens/strains), by taking extreme care to avoid sample contamination, and by systematically and blindly running appropriate negative and blank controls.

To be further assured that these precautions have worked, one can use the “multiple detection-state” models of Miller and colleagues [15] in a first round of analyses to check that the false-positive rate (*p*_10_) is suitably low (say, *p*_10_ < 0.02, for a specificity >0.98)—or, alternatively, that a model with *p*_10_ fixed at 0.0 (i.e., explicitly assuming 100% specificity) performs about equally well (as judged by, e.g., AICc scores differing by <2.0 units) as one in which *p*_10_ is estimated from the data. To fit “multiple detection-state” models [15], however, at least some of the detections must be unambiguous—for example, and as mentioned above, definite identification of the pathogen in a microscope slide (see, e.g., [8]). If this analysis suggests that *p*_10_ is too high (i.e., specificity is too low), one might consider either repeating the study (at least, retesting some samples) under more stringent protocols or reporting the results of the “multiple detection-state” models. In this latter case, it is important to remember that “sensitivity” estimates from these two-level (subject, test) models are in reality estimates of *p* × θ, so that they can only be interpreted as “test sensitivity” by assuming that sample-level availability is perfect (θ = 1.0). Three-level models (subject, sample, test) allow us to relax this strong assumption—at the cost of assuming 100% specificity [13].

## Applying the approach, with a hypothetical example

I have briefly outlined an approach to tackling a problem that is inherently hierarchical with a similarly hierarchical statistical modeling framework. The probabilities of *subject-level infection*, *sample-level availability*, and *test-level detection* all have a bearing on the results that doctors see and use to make decisions (Figs 1 and 2). To apply this approach, one has to be aware of the need for replication at all levels of the hierarchy (but note that the models naturally deal with missing test results [9,10]) and of the importance of avoiding false positives [13,14]. Both replication and quality control are already common in clinical research—replicate tests are typically run on samples (often also replicated) from multiple subjects, and good laboratories adhere to best practices aimed at avoiding false positives (see, e.g., [16]). Clinical research teams, then, just need to take one further step ahead, as many ecologists [9,10,13–15] including disease ecologists [8,17,18] have done already, to get formally hierarchical about their naturally hierarchical problems. Back to Chagas disease diagnosis and cure assessment, I think that clinical or epidemiological researchers like Alonso-Padilla and colleagues [1] would greatly benefit from having reliable estimates of subject-level Ψ, sample-level θ, and test-level *p*; from knowing how they vary with subject-, sample-, or test-level covariates; and from getting, for each estimate, a measure of uncertainty.

Imagine, for example, a situation in which modeling data from a group of subjects with untreated, chronic Chagas disease (Ψ = 1.0) yields estimates of θ ≈ 0.7 for the average availability of *T*. *cruzi* DNA in single blood samples and *p* ≈ 0.85 for the average sensitivity of single PCR runs. This tells doctors that, to get an average probability >0.95 that *T*. *cruzi* DNA is available for detection, they need to draw samples on 3 or more independent occasions, so that θ_3s_ = 1–(1–0.7)^3^ = 0.973; 2 occasions would yield θ_2s_ = 1–(1–0.7)^2^ = 0.910. Knowing that the test has, on average, *p* ≈ 0.85 allows doctors to calculate how many test replicates they need to run to get a “combined” *p* > 0.95. If the sample contains *T*. *cruzi* DNA (θ = 1.0), then 2 tests will do: *p*_2t_ = 1–(1–0.85)^2^ = 0.978. Yet doctors now know that θ ≈ 0.7, not 1.0, and also that pooling samples drawn on 3 occasions will yield θ_3s_ = 0.973; 2 replicate tests on the pooled sample will then miss only about 5% of infections: *p*_2t_ × θ_3s_ = 0.978 × 0.973 = 0.951. Using 1 sample drawn on 1 occasion, so that θ ≈ 0.7, the best our doctors can hope for is to reach a 0.7 probability of detecting the target—and they would need at least 4 replicate tests (each with *p* ≈ 0.85) to get above 0.699 on average.

This very simple example shows how this approach can help doctors make better strategic decisions—in this case, they should choose “replicate sampling occasions, sample pooling, and limited test replication” instead of the largely useless “multiple test replication on single-occasion samples.” A “single-occasion sample, 2 test replicates” strategy (see [1]) would be all but hopeless in this scenario—about 31.6% of infections would be missed, most of them because *T*. *cruzi* DNA was simply not available for detection in the sample.

Now suppose that the patients are treated with benznidazole; this lowers parasitemia, and modeling posttreatment data reveals that single-sample θ has dropped from ~0.7 to ~0.5, whereas single-PCR sensitivity has remained unchanged at *p* ≈ 0.85. Now one can ask, “What would be the best strategy to get informative and reliable data about *T*. *cruzi* presence (Ψ) in benznidazole-treated subjects?”

So far, the results of this (reasonably realistic, I think) hypothetical example look quite disheartening. But now suppose that a different test is designed whose *target for detection* is not *T*. *cruzi* DNA but a (highly specific) biomarker that circulates more widely in the bloodstream of infected subjects—say, with average availability θ ≈ 0.9. Then, a “single-occasion sample, 2 test replicates” strategy (with sensitivity *p* ≈ 0.85 as above) would miss about 12% of infections, and a “2-occasion pooled sample, 2 test replicates” strategy would miss just over 3.2%. Taken together, then, results resembling those of my example would clearly suggest that *efforts should be directed toward developing tests based on targets with high θ*. Molecules with high θ values in easy-to-sample tissues or bodily fluids would be particularly interesting—with, e.g., saliva or urine preferable to blood, and capillary blood preferable to venous blood [1]. Among such widely bioavailable targets, parasite-derived biomarkers (e.g., excreted-secreted antigens [4], proteins or nucleic acids in extracellular vesicles [5], or small RNAs [19]) can in principle be expected to yield overall higher test specificities than host-derived molecules such as anti-*T*. *cruzi* antibodies.

## Conclusion, with some caveats

I expect that this Viewpoint will spark interest in a modeling framework that closely mirrors, and thus helps dissect, the hierarchical problem of classifying subjects as infected or not with a pathogen—and of doing so, in addition, based on observations that result from imperfect sampling and testing procedures. The approach is a potentially powerful addition to the clinical-epidemiological research toolkit. It can, in particular, sharpen our view of how diagnostic and test-of-cure methods actually perform—a view that, ultimately, lies at the heart of clinical decision-making and epidemiological inference. The models are easy to fit in the free software Presence [20], and Bayesian implementations are also available (e.g., [13,14,18]). To capitalize on this approach, users will need both adequate data (with emphasis on replicate sampling/testing and quality control) and a solid understanding of the technical and biological subtleties of the problem at hand. For example, “target availability” really means “target availability *above the test’s limit of detection*” and may depend on subject-specific and time-varying pathogen loads or on sample-processing protocols [6,7,17,18]; different biomarkers, moreover, may be “markers” of different things—including, for some antibody/pathogen combinations, markers of pathogen *absence* from a once-infected host. I, therefore, do not mean to suggest that the models will always or automatically get us the right answers—just that, as the dictum has it, they are bound to be helpful.
